# Frequency of complications of falling from the walnut tree, as an occupational-seasonal injury

**DOI:** 10.5249/jivr.v10i2.980

**Published:** 2018-07

**Authors:** Tooba Hoseini Azizi, Sima Sadat Hejazi, Ahmad Kameli

**Affiliations:** ^*a*^Faculty of Nursing and Midwifery, North Khorasan University of Medical Sciences, Bojnurd, Iran.

**Keywords:** Accidental fall, Walnut tree, Occupational injury

## Abstract

**Background:**

Falling from a tree is one of the major causes of serious injuries in farmers but it can be prevented. Walnut is one of the most important agricultural products in Iran and falling from walnut trees is common during the season of harvest. The aim of this study was to determine the frequency of complications due to falling from walnut tree in patients referred to the emergency department of Imam Ali Hospital in Bojnurd City, Iran.

**Methods:**

A descriptive cross sectional study was conducted on 127 patients with injuries due to falling from the walnut tree in Bojnurd City, Iran, in the walnut harvest season of August 2013 to November 2014. The tools used for data collection in this study included a demographic information form, checklists of information about the injury and the follow-up checklist of treatment. Data were analyzed using descriptive statistics by the SPSS software version 16.

**Results:**

From a total of 127 injured patients, 120 cases (94.4%) were males with a mean age of 36.49 ± 15.65 years. Five patients died on the day of admission. Eighty two patients (64.56%) were admitted in hospital wards. The most common type of injuries were trunk injuries (26.19%), followed by spinal cord injuries (18.1%). Also, 7 patients suffered from complete spinal cord injury.

**Conclusions:**

Injuries caused by falling from walnut trees are seasonal and impose large financial burden on our health system. In most cases, young men suffer from varying degrees of disability and experience financial problems. It is very important to train farmers and workers, so as to prevent such injuries.

## Introduction

Falling from a height is one of the major causes of fatal and non-fatal injuries in adults. It usually occurs while working, as an occupational accident and results in serious but preventable injuries.^1,2^

The severity of injuries which result from falling depends on the height, direction of fall, landing surface and the patient's age. The most vulnerable parts are the head, spine, and limbs.^2^

Falling from trees has been widespread among farmers who climb trees to harvest products and has led to vast debilitating vascular and spinal injuries.^3-5^ The injuries caused by falling from a tree in mechanized agricultural settings is significantly lower than in the unmechanized agricultural setting. The use of tree stands, with safety equipment, has reduced the amount of damage caused by falling from trees.^4,5^

Walnut trees are one of the most important poles of the agricultural economy. Walnut trees are one of the highest trees (15 to 30 meters high) with a long life, its fruits grow at the end of its long branches and have a slippery surface. To harvest walnuts, farmers must climb the tree to pick the fruits with their hands.^1,3,6^

The ripened walnut fruit becomes loose at its base and falls, but this can cause product loss or damage. On the other hand, the tendency to collect green walnut encourages the growers to choose quick, economic, but risky ways to harvest the product.^2^

One of the main problems of the farmers is falling from the high point of such trees, which occurs due to the presence of wormeaten branches, unpruned trees, and recklessness of some workers during walnut harvesting.^2,6^

There are many old and natural sites of walnut trees which still bears fruit in Iran. The good price of walnut in domestic markets is the reason why farmers take the risk of climbing to harvest the fruits.

Due to the high altitude of walnut trees in Iran, falling from such trees can cause many problems such as back trauma, fractures in the upper and lower limbs, heads, multiple trauma, spinal cord injury and death, which in turn imposes health costs. North Khorasan Province is one of the natural sites of walnut trees in Iran. In this study, information about the frequency and types of traumas caused by falling from walnut trees was studied, in North Khorasan province, Iran.

## Methods 

This prospective descriptive study was held in the trauma emergency department (ED) of the university hospital in Bojnurd, North Khorasan, Iran. All the patients admitted for injuries due to falling from walnut trees between August 2013 to November 2014 were included in the study. 

**Setting**

Imam Ali University Hospital, Bojnurd, is the only equipped trauma center in the province where all patients are referred to. 

**Data collection and analysis**

The data collection tools included a demographic information form, checklists of information about the injury and the follow-up checklist of treatment. Triage nurses filled the demographic form and the checklist of information related to the injury. Also, this study recorded results of the physical examination, radiography, treatments, and patient’s outcome, duration of hospitalization and their follow-up costs. Followed-up was conducted on patients who had a medical profile, after three months of discharge. 

All subjects signed a written informed consent before the study began and were free to withdraw from the study at any time during the study. Data was encoded and analyzed using IBM SPSS statistics version 16. 

## Results

Between August 2013 and November 2014, a total of 127 patients were referred to the trauma center after falling from the walnut tree. 

**Demographic and time features**

One hundred and twenty patients (94.4%) were males and aged between 9 to 75 years old with a mean age of 36.49 ± 15.65. Sixty one cases (47.24%) were farmers and workers and 107 cases (84.25%) had insurance. Most patients (74.8%) were admitted in September and October and were mostly referred to the hospital in the evening shift (69 people, 54.33%). Only 27.56% (n = 35) of the cases were transferred to the trauma center by the emergency services while the rest were transported to the hospital by family members. 

**Mechanisms of injury**

The most common cause of fall was the breaking of branches (58 cases, 45.66%) while the average height of the fall was 2.36± 4.02m (1-14 m). 

A total of 93 patients were hospitalized and 29 cases were treated as out-patients. Five patients died on the day of admission, 11 patients were discharged from the emergency ward after ensuring normal results in X-Ray, abdominal sonography and brain CT scan, and 82 cases (64.56%) were admitted in hospital wards. 

Among the outpatients, 17 cases with skin laceration were treated by wound care (suture and dressing).

All the patients who died had traumatic brain and skull injuries.

Most cases had the trunk injury (32 people, 26.19%) (Table 1).

**Table 1:** Patterns of injury (127 cases).

Injury	Number (%)
**Head Injuries**	5 (3.93)
**Spinal Injuries**	23 (18.11)
Cervical	5 (3.93)
Thoracic	10 (7.87)
Lumbosacral	8 (6.3)
**Upper Limb Injuries 15(11.81)**	15(11.81)
**Lower Limb Injuries 26(20.47)**	26(20.47)
**Trunk Injuries**	32(25.19)
Chest	7(5.51)
Pelvic	15(11.81)
Abdomen	10(7.9)
**Head and Face Laceration**	17 (13.38)
**Combined Injuries**	9 (7.08)
Limb and pelvic	5 (3.93)
Pelvic and chest	3 (2.36)
Pelvic and chest and lumbar spine	1 (0.78)
**Total**	127 (100)

Ten patients (7.9%) had abdominal trauma, 3 patients underwent splenectomy and 2 patients with penetrating abdominal trauma were treated surgically. In other cases, where sonography results showed the existence of free fluid in the abdomen, a diagnostic laparotomy was performed. There were 2 cases of liver hematoma and 2 cases of renal hematomas and one healthy case.

Among patients with spinal trauma, 15 cases underwent surgical treatment for decompression and stabilization procedures and in 5 cases closed reduction was performed. 

Most limb fractures (30 cases) were managed non-surgically by closed reduction and casting. Skeletal traction and internal fixation were done in 11 patients.

**Outcomes**

Seven patients (5.51%) with spinal injury had neurological defect at the time of discharge while 3 patients had spelenectomy. The remaining 75 patients were discharged with good health condition.

No significant relationship was found between the height of the fall and the type of injury (p= 0.80, df=9) and the cause of the fall and type of injury (p=0.188, df=9).

The duration of hospitalization was from 1 to 22 days with a mean of 3.12 ± 4.51 days (Table 2).

**Table 2:** Demographical and clinical characteristics of the patients.

Characteristics	Number (%)
**Age, y**	
Mean (± standard deviation)	35.42 ± 16.54
**Sex**	
Male	120(94.4)
Female	7(5.6)
**Job**	
Farmer	41(31.5)
Worker	20(15.74)
Other	66(51.96)
**Location**	
Village	80(63)
Town	47(37)
**Insurance Status**	
Yes	107(84.25)
No	20(15.74)
**Transported by**	
Family members	92(72.9)
Emergency medical system	35(27.56)
**Emergency Admission Shift**	
Morning	34(6.77)
Evening	69(54.33)
Night	24(18.89)
**Month of admission**	
August	25(19.68)
September	42(33.07)
October	53(41.73)
November	7(5.51)
**Cause of Falling**	
Slipping	26(20.47)
Branch breaking	58(45.66)
Loss of balance	11(8.66)
Lightheadedness	12 (9.44)
Unknown	20(15.74)
**Survey**	
Expired	5(3.93)
Admission in wards	82(64.56)
Admission in emergency	11(8.66)
Outpatient and referred	29(22.83)

About 28000 US $ have been spent on their care and treatment. 

In-patients who were admitted with medical records follow-up continued 3 months after discharge. Three months after discharge, 36 patients (28% of all cases and 44% of hospitalized cases) were unable to return to their job (school) and were treated at home. 

## Discussion

In this study, the epidemiological situation of injuries after falling from walnut trees was studied for the first time in North Khorasan. The charges of these injuries and follow-up of the patients were conducted for the first time in Iran. 

According to the FAO in 2011, Iran is the second largest producer of walnut after China with a production of 485000 tons per year.^7^

North Khorasan Province is one of the most important centers of planting walnut in Iran.

There are about 700000 walnut trees in an area of 2120 ha in North Khorasan. A total of 81,600 people have agricultural activity in this province, with an average age of 48 years and an annual salary of $ 6,500 to $ 7,000. About 40% of the farmers are illiterate and about 38000 of them are involved in fruit harvesting.^8^ Because walnut planting expanded around the orchards and mixed with other fruits trees, it is difficult to estimate the number and characteristics of the population at risk. The walnut harvest season is from mid-August to November and walnut is an important source of income for farmers in NK province. 

The results of the present study showed that the majority of the injured patients were young men from rural areas who fell down following the fracture of a walnut tree branch and from a height of more than 4 m onto a rocky ground.

Falling from trees is the cause of most fall-related injuries.^9-15^ In countries such as Iran, where the lives of most of the rural areas population is dependent on fruit trees, the main reason behind falling from trees is the method of nuts collection. Workers and farmers stand on the branches of trees and use a stick to collect the nuts. In this way, it is possible for the person to lose his balance or fall down due to the slippery surface of the branch.^5,6,16,17^ The leopard moth is the main pest of walnut trees in Iran. Pollution with leopard moth pesticides in walnut trees (Zeuzera pyrina L.) produces a superficially healthy and thick but hollow branch and may break as a result of the heavy weight of the human body. ^18^ In this study, breaking branches were reported as the reason for half of the falls. While slipping was reported as the cause of most falls from other fruit trees.^5^

In the present study, as well as the results of other studies, most of the people that fell from trees are males.^1-3,6,17^ This is because men are responsible for the collection of fruits from trees.

It was found that most of the patients were young with an average age of 35 years, this finding is consistent with the results of other studies.^1-3,6,16,17^ Also, it was found that half of the injured patients were between 20 and 50 years whereas 23.6% were over 50-years old. Ersoy et al. recorded the same results.^16^ The young workers collected fruits to pay for their living expenses. Disabling injuries caused by falling from trees can cause a negative economic impact. 

Falling from a height over 15 m will cause serious injuries.^4^ The Persian walnut trees grow to a height of 15 to 40 m. ^6^ In this study, 3.93% of the injured (5 people) were admitted with no vital signs due to brain injury and skull fracture. Baba et al. showed a mortality of 3.36% in 5-years in India, which was similar to our study. ^6^ In the only study on the fall from walnut trees in Iran published by Javadi et al., the mortality rate was reported as 10% (5 patients). ^1^ The highest rate of mortality reported after a fall from walnut trees is 24.1%.^2^ The reason for the lower mortality rate in our study compared to two recent studies is due to the fact that a number of injured patients died immediately after the fall and were not referred to the hospital. Also, due to the proximity of our traumatic site with walnut gardens, injured people arrived at the hospital in a short time and were saved. Also, as a result of the easy access to our trauma center and having a proper road, it was easier to treat and rescue patients promptly.

Falling from a high altitude can cause serious spinal cord injuries or fatalities. If the direction of fall is on the face or forehead, the cervical vertebrae is injured due to hyperextension of the neck.^19^ In this study, two c4 fractures and three c3-c4 protrusions were seen. Three months after discharge, it was revealed that except in one case of the protrusion cases, the remaining patients were unable to return to work and are still under treatment. There was complete spinal cord injury at the level of the lesion. In comparison with similar studies, spinal cord injury was less observed in the current study. While nearly half of the number of people who fell from the walnut trees suffer from spinal cord injuries due to falling from a high altitude,^2,3,6,16^ only 18% of the injuries in this study were related to the spinal cord. This difference may be due to the lack of precise investigation of missed cases. The main cause of fall in this study was the breaking of walnut tree branches which led to the fall of the branches on the legs and lower extremity injuries. Differences in soil bed under the tree could have an impact on the severity of the injury.^13^ In the study area, the soil is less rocky and mainly covered with the soil and plants. 

Another high-risk complication of falling from a height is abdominal trauma.^11^ The types of abdominal injury depend on the location of landing on the ground or collision of the belly with the branches. Spleen and liver showed the highest injury rates after blunt trauma.^17^ It was found that 3 of 10 patients with abdominal trauma, underwent splenectomy and 2 were treated with liver hematoma in this study. Wani et al. examined abdominal trauma in the injured people who fell from walnut trees in a 5-year period. Of 17 cases with abdominal trauma, 8 cases underwent splenectomy ^18^ Tabish et al. reported the splenectomy of 7 out of 15 patients with abdominal trauma. ^2^ It was found that the lower extremity is injured 2 times more than the upper extremity, and most of the upper extremity fractures were observed at the distal radius and clavicle (11 of 15 cases of upper extremity trauma). This is due to the openness of hands during the fall. In the lower limb, most injuries occurred in the ankle (12 of 26 cases) in which landing on the foot increased the injury. In all the studies on fall from trees, lower extremity injuries were observed more than the upper ones. ^2,3,6,11,12,14,16^

Some studies reported that an imbalance due to alcohol abuse can be considered as the cause of fall.^12^ Since alcohol consumption is not common for religious reasons among Muslims living in the area under the study, alcohol does not have an effect on the injuries in this study. Environmental heat may be another cause of light headedness and fall.^20^ Since the temperature in our province in the walnut harvest season (September to November) is moderate (mean temperature is 26°C in September to 16°C in November)^21^ the heat factor cannot be effective in falling of the tree.

The majority (73%) of cases were not transferred to the hospital by EMS. Failure to transfer patients with safe methods, especially those with spinal cord injuries, worsened the complications caused by the fall. The general awareness that an injured person must be transferred to the hospital by the EMS is necessary. Immobilization until the arrival of the emergency can reduce the severity of injuries caused by the fall.

Information on the two categories of cases is not available to us. 

The first group consisted of those who had superficial injuries and were treated at home, while the second consisted of those who died on the scene and were not referred to the hospital.

A follow-up was conducted for patients with medical records after three months. Nearly a third of all cases and half of the hospitalized cases were not able to return to work after three months. All patients were male and were family income providers. Inability to work and the cost of treatment have a lot of financial burden on the family.

In conclusion, injuries caused by a fall from walnut trees are seasonal and impose large financial burden on our health system. Most cases are young men who suffer from varying degrees of disabilities and face economic problems in meeting the cost of living. Training farmers and workers is very important to prevent such an injury. Using tree stands and anti-slip boots and helmet, chest and abdomen covers along with the treatment of leopard moth and systematic pruning of high branches can reduce the severity of injuries. Preventive education in schools in rural areas is also recommended. 

This study had some limitations. Data were extracted from hospital records and it is a case for underestimating the frequency of falling attributed to walnut tree injury. 

**Acknowledgments**

The authors gratefully acknowledge the contribution of all patients who participated in this study and gratefully thankful to all nurses who gathered the data and physicians of emergency unit of Bojnurd Imam Ali Hospital. 
